# Modulation of adipocyte differentiation by omega-3 polyunsaturated fatty acids involves the ubiquitin-proteasome system

**DOI:** 10.1111/jcmm.12194

**Published:** 2014-01-29

**Authors:** Cezary Wójcik, Kimberly Lohe, Chenzhong Kuang, Yan Xiao, Zeida Jouni, Eduard Poels

**Affiliations:** a Department of Family Medicine, Oregon Health and Science UniversityPortland, OR, USA; b IU School of MedicineEvansville, IN, USA; c Mead Johnson NutritionEvansville, IN, USA; d DSM Nutritional ProductsColumbia, MD, USA

**Keywords:** DHA, DPA, EPA, ω-3 polyunsaturated fatty acids, ubiquitin, proteasome, fatty acid synthase, 3T3-L1 cells

## Abstract

We have evaluated the effects of three different omega-3 polyunsaturated fatty acids (ω-3 PUFAs) – docosahexaenoic acid (DHA), eicosapentaenoic acid (EPA) and docosapentaenoic acid (DPA) on fat accumulation and expression of adipogenic and inflammatory markers using both 3T3-L1 pre-adipocytes and differentiated 3T3-L1 adipocytes. Our results indicate that ω-3 PUFAs induce the degradation of fatty acid synthase through the ubiquitin-proteasome system, which is likely to have beneficial metabolic effect on adipose cells. Omega-3 PUFAs also increase overall levels of polyubiquitinated proteins, at least in part through decreasing the expression of proteasome subunits. Moreover, adipocytes are resistant to proteasome inhibition, which induces adipophilin while decreasing perilipin expression. On the other hand, ω-3 PUFAs decrease expression of SREBP1 while inducing expression of adipophilin and GLUT4. Moreover, all three ω-3 PUFAs appear to induce tumour necrosis factor-α without affecting NFκB levels. All three ω-3 PUFAs appear to have overall similar effects. Further research is needed to elucidate their mechanism of action.

## Introduction

Only two fatty acids are bona fide essential for humans: α-linolenic acid (ALA) and linoleic acid (LA). In contrast, ω-3 polyunsaturated fatty acids (ω-3 PUFAs), which include docosahexaenoic acid (DHA), eicosapentaenoic acid (EPA) and docosapentaenoic acid (DPA), are not essential as they can be produced from alimentary ALA. However, the capacity of converting ALA to ω-3 PUFAs is limited. Eicosapentaenoic acid and DPA can be interconverted. Docosapentaenoic acid serves as the precursor of several active metabolites, and additionally, can be further converted to DHA. However, neither DPA nor EPA can be produced from DHA. Typical Western diet is usually quite poor in ω-3 PUFAs, which are enriched in marine food sources.

Supplementation of high-fat diet in laboratory animals with ω-3 PUFAs decreases weight 1–3. In humans, there is a significant inverse correlation for body mass index (BMI) *versus* plasma levels of total ω-3 PUFAs 4. Diet enriched in ω-3 PUFAs can lead to weight loss 5. In addition, ω-3 PUFAs reduce cardiovascular risk associated with obesity and metabolic syndrome 6 as well as have anti-inflamamtory, anti-apoptotic and neuroprotective activities 7. Anti-inflammatory effects are beneficial in asthma, ulcerative colitis and arthritis 8.

Omega-3 PUFAs exert their biological effects through multiple mechanisms. Much of their biological activity has been attributed to the activation of a cell surface receptor GPR120 9. However, they may also interact with other receptors (*e.g*. PPARγ, α and δ), alter lipid rafts and mitochondrial membranes, affect eicosanoid metabolism, and activate AMP-activated protein kinase as well as other signal transduction pathways 10.

Adipocyte differentiation is a complex process, which can be reproduced *in vitro* using established cell lines such as 3T3-L1 adipocytes. It involves an interplay of pro-adipogenic transcription factors such as SREBP1 and PPARs, expression of adipogenic proteins such as fatty acid synthase (FAS) and the lipid droplet associated proteins perilipin and adipophilin, production of cytokines and adipokines [such as tumour necrosis factor (TNF)-α, adiponectin and leptin] as well as activation of intracellular signalling pathways, including pro-inflammatory and pro-survival NF-κB signalling, unfolded protein response associated with up-regulation of the endoplasmic reticulum (ER) chaperone BiP as well as pro-apoptotic FAS signalling, leading to activation of caspases 11.

The ubiquitin-proteasome system (UPS) degrades both short-lived regulatory proteins, such as cyclins and transcription factors, as well as long-lived structural proteins 12. The UPS has a hierarchical structure, where a single E1 or ubiquitin-activating enzyme, couples itself with a dozen of E2s or ubiquitin conjugating enzymes, followed by over 500 different E3s or ubiquitin ligases, many of them formed by multisubunit complexes 12. Once polyubiquitinated, proteins are doomed for degradation through the 26S proteasomes. They are barrel shaped assemblies of four stacked rings (20S proteasomes) with an activator complex (PA700 or 11S) attached to its ends. While the proteolytic activities reside within six subunits of the 20S core, it is the PA700 particle, which harbours machinery necessary to recognize polyubiquitinated substrates, remove the ubiquitin moieties and feed the unfolded polypeptide chain into the central chamber inside the 20S core 13,14.

Pharmacologic inhibition of proteasomes blocks adipose differentiation of both murine pre-adipocytes 15 and human adipose-derived stem cells when applied early in the differentiation process, when proteasome activity is at its peak 16. Down-regulation of proteasome subunits by RNA interference inhibits adipocyte differentiation 17. This effect likely depends on the regulation of transcription factors, many of them involved in regulation of adipogenesis, as well as crucial proteins directly involved in adipogenesis. For example, proteasomes are known to degrade adipophilin during adipose differentiation mechanism 18 as well as PPARγ upon ligand binding 19. However, while proteasome inhibition in rats significantly reduces lipogenesis 20, transgenic mice with impaired proteasome function develop obesity and hepatic steatosis 21. The reason of that discrepancy is unclear, indicating that our understanding of the role of the UPS in adipose differentiation is still very limited.

Omega-3 PUFAs have anti-adipose activity associated with induction of mitochondrial biogenesis, up-regulation of adiponectin expression, and a decrease in inflammation within the adipose tissue 22,23. Supplementation of high-fat diet with ω-3 PUFAs decreases weight of experimental animals 1–3. In humans, there is a significant inverse correlation for BMI *versus* plasma ω-3 PUFAs 4. Diet enriched in ω-3 PUFAs can lead to weight loss 5. No systematic studies have been performed comparing the effects of all three ω-3 PUFAs on differentiating adipocytes. The objective of this work was therefore to study the effects of DHA, DPA and EPA on 3T3-L1 adipocytes at different stages of differentiation, using as control a saturated fatty acid (stearic acid, SA) as well as an ω-6 PUFA, LA. In particular, we analysed their effects on multiple signalling pathways involved in adipogenesis.

## Material and methods

### Free fatty acids

Free fatty acids (FFAs; Sigma-Aldrich, St. Louis, MO, USA) were dissolved in USP Grade ethanol to obtain a 10 mM stock, aliquoted, and frozen at −70°C until used. Free fatty acids (Matreya, Pleasant Gap, PA, USA) have been purchased as 5 mg aliquots under inert gas. They were kept frozen at −70°C and dissolved in USP grade ethanol to a stock concentration of 10 mM prior to addition to media, reaching a final 100 μM concentration. Once added to culture media, the media were used within 48 hrs.

### Reagents

MG132 (Calbiochem, La Jolla, CA, USA), troglitazone and GW9662 have been prepared as 10 mM stocks in DMSO and kept frozen at −20°C until added to media (final 10 μM concentration). Tunicamycin (Calbiochem) has been prepared as a 10 mg/ml stock in DMSO and kept frozen at −20°C until added to culture media (final 20 μg/ml concentration). Tiron has been prepared as a 2 mM stock in sterile ddH_2_O and kept frozen at −20°C until added to culture media reaching a final 20 μM concentration. Reagents were from Sigma-Aldrich unless otherwise indicated.

### Antibodies

For Western, the following antibodies were used: adipophilin (1:10,000, guinea pig) and perilipin (1:100,000, guinea pig) from Fitzgerald Industries International (Acton, MA, USA); CHOP (1:200, rabbit), SREBP1 (1:200, mouse), NFkB (1:200, mouse), PPARγ (1:200, rabbit), GLUT4 (1:200, mouse) from Santa Cruz Biotechnology (Santa Cruz, CA, USA); anti-KDEL (BiP, 1:2500, mouse) from MBL International (Woburn, MA, USA); anti-caspase 3 (1:1000, rabbit) from Cell Signaling Technology (Danvers, MA); FAS (1:3000, mouse) from BD Transduction Laboratories (Franklin Lakes, NJ, USA); actin (1:10,000, rabbit), and polyubiquitin (1:500, rabbit) from Sigma-Aldrich; Hsp70 (1:1000, mouse) from Stressgen Bioreagents (Victoria, BC, Canada), while anti-β1 (1:5000 rabbit) and anti-S4 (1:2000, rabbit) antibodies were custom obtained 24. Secondary antimouse, anti-rabbit and anti-guinea pig Fab' fragments labelled with horseradish peroxidase (HRP) or alkaline phosphatase (AP) were from Jackson Immunoresearch (West Grove, PA, USA).

### Cell culture

3T3-L1 fibroblasts (ATCC, Manassas, VA, USA) were propagated in DMEM (Invitrogen, Grand Island, NY, USA) with 10% FBS (Atlanta Biologicals, Lawrenceville, GA, USA), split 1:6–1:8 before 80% confluence. Once the cells reached 100% confluence in poly-l-lysine-coated plates, they were maintained for 2 days in growth media (DMEM, 10% FBS), which were replaced with full differentiation media (growth media + 1 μg/ml insulin, 0.5 mM IBMX, and 1 μM dexamethasone) for next 2 days (D0 and D1). Cells were maintained in insulin media (growth media + 1 μg/ml insulin) for up to 8 days (D2–D10). If not indicated, reagents were from Sigma-Aldrich. Samples were collected at D0, D2, D4, D6, D8 and D10. MG132 was added at D3 until D7. FFAs with different inhibitors were incubated for 24–48 hrs using D0 pre-adipocytes or D3 adipocytes.

### Oil red O measurement

For oil red O (ORO) staining, cells were supplemented with different FFAs and cultured for 24 or 48 hrs (collected at D1 or D2). Cells were washed with PBS, fixed in 10% formaldehyde, washed, air dried and stained with freshly prepared ORO working solution was added. Oil red O working solution was prepared filtering six parts of 0.35% ORO (Harleco, Philadelphia, PA, USA) stock in isopropanol and four parts ddH_2_O. After 10 min. of incubation, solution was aspirated, cells were washed, and air dried. Oil red O was eluted with 100% isopropanol and transferred to cuvettes. Optical density (OD) 500 nm was read by DU 640 spectrophotometer (Beckman Coulter, Brea, CA, USA). Data obtained from three different experiments were used for analysis.

### MTT Assay

3-(4,5-dimethylthiazol-2-yl)-2,5-diphenyltetrazolium bromide (MTT) cytotoxicity assay was done as described before 25. MTT assay measures mitochondrial activity in metabolizing cells and therefore can be used as an approximate measurement of cell number and viability. At 24 or 48 hrs of incubation, MTT solution was added to all wells and mixed gently. MTT stock in PBS was prepared freshly. After 4 hrs, media were aspirated and discarded. Isopropanol was added to each well. After 10 min. on a rocker and a gentle vortexing, samples were transferred to disposable cuvettes. OD 570 nm was read at using DU 640 spectrophotometer (Beckman Coulter). Data obtained from three different experiments were used for analysis.

### RT-PCR

RNA was isolated from cells using Trizol (Invitrogen, Carlsbad, CA, USA). Semiquantitative RT-PCR was performed with the OneStep kit (Qiagen, Valencia, CA, USA) using pairs of primers (IDT DNA, Coralville, IA, USA; Table 1). DNA electrophoresis was performed on 1% agarose gels using 100 bp molecular size markers (Fisher Scientific, Waltham, MA, USA). DNA was labelled with ethidium bromide. Images were acquired using Kodak Image Station 4000MM (Eastman Kodak, Rochester, NY, USA). Each experiment was repeated three times and the representative gels were used for illustration purposes.

**Table 1:** Primers used for semiquantitative RT-PCR

Gene	Accession no.	Forward primer	Reverse primer
Adiponectin	NM_009605	AGGCATCCCAGGACATCCT	TCGTAGGTGAAGAGAACGGC
TNF-α	NM_013693	ATGAGAAGTTCCCAAATGGC	TTGACGGCAGAGAGGAGGTT
FAS	NM_007988	ATAGCCGGTATGTCGGGGAA	AGTGAGGCTGGGTTGATACCT
Actin	BC099371	TTCCTTCCTGGGCATGGAGT	ATCCACATCTGCTGGAAGGT

### SDS-PAGE and Western blotting

Whole cell lysates were obtained in radioimmunoprecipitation assay buffer supplemented with Complete Mini™ protease inhibitors (Roche, Mannheim, Germany) and sodium orthovanadate. Samples were normalized to the same protein concentration determined using Bradford reagent (Bio-Rad, Hercules, CA, USA). SDS-PAGE was done using the MiniProtean II system (Bio-Rad). Western blotting was performed on Immobilon-NC membrane (Millipore, Billirica, MA, USA). Primary antibodies were detected with secondary AP or HRP-conjugated Fab' antibody fragments (Jackson Immunoresearch). Horseradish peroxidase was detected using the Amersham ECL™ Advance kit (GE Healthcare, Piscataway, NJ, USA) and images were acquired using Kodak Image Station 4000MM (Eastman Kodak). Alkaline phosphatase was detected by the one Step NBT/BCIP developer (Fisher Scientific). EZ-Run™ protein standards (Fisher Scientific) have been used as molecular weight markers. Each experiment was repeated three times and the representative gels were used for illustration purposes.

### Statistical analysis

Each experiment was repeated at least three times. Data from MTT assays and oil red O staining were analysed using Student's *t*-test and expressed as mean ± SD. *P*-value has been indicated on the respective figure.

## Results

### Toxic effects of ω-3 PUFAs

**Figure 1:**
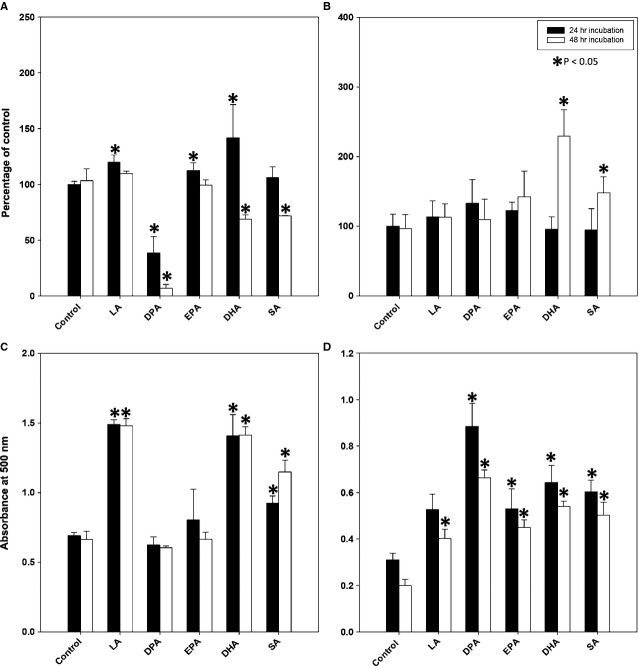
Variability in the effects of selected free fatty acids (FFAs) depending on their source, Sigma-Aldrich (A and C) *versus* Matreya (B and D). MTT assay shows cytotoxicity of docosahexaenoic acid and docosapentaenoic acid from Sigma-Aldrich (A) but not from Matreya, where they appear to stimulate growth (B). Oil red O extraction shows pro-adipogenic effects of FFAs from Matreya (D), but not from Sigma-Aldrich (C). Each experiment was repeated three times, results are expressed as mean ± SD.

**Figure 2:**
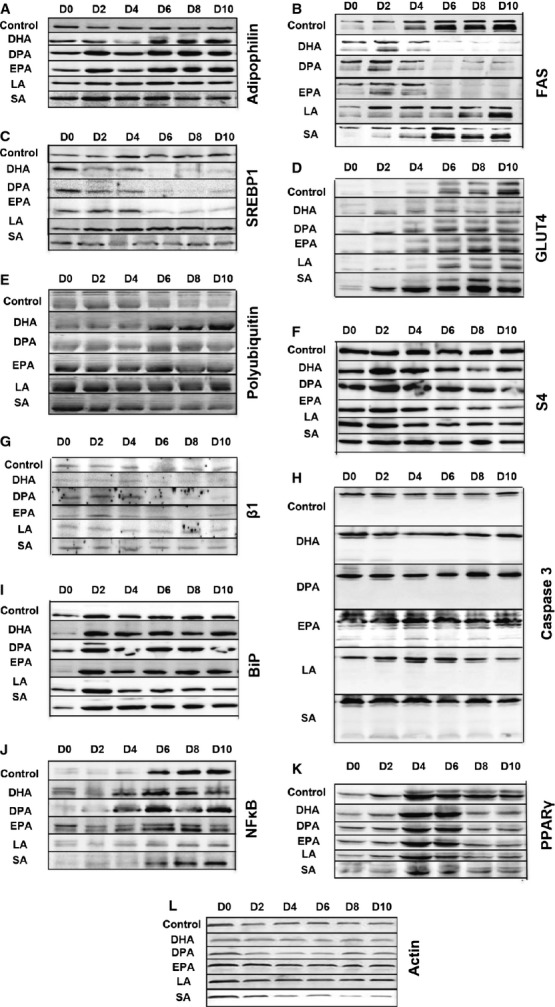
Western blots showing the effects of a long incubation of the different free fatty acids with 3T3-L1 adipocytes undergoing differentiation. Cells were harvested every 2 days, from D0 until D10. Westerns are grouped by the same antibody indicated on the right, while different treatments are indicated on the left of each figure. Figures are representative of three experiments.

Docosahexaenoic acid and DPA from one manufacturer (Sigma-Aldrich) caused widespread cell death as early as within 24–48 hrs of incubation as reported by others 26. However, another batch of reagents from the same manufacturer revealed differences in response. The supplier acknowledged that the purity of its ω-3 PUFAs from heavy metals and other contaminants could not be guaranteed. Subsequently, experiments were performed with FFAs provided by a different manufacturer (Matreya), guaranteed to be free from toxic contaminants. In contrast to DPA and DHA from the first manufacturer, DPA and DHA from the second manufacturer did not induce cell death as estimated by the MTT assay (Fig. 1A and B). There was no evidence of caspase-3 cleavage even after 10 days of incubation (Fig. 2H), while caspase-3 cleavage appeared as early as within 24 hrs of incubation with DPA and 48–72 hrs of incubation with DHA from Sigma-Aldrich (data not shown). Stearic acid, LA and DHA from either Sigma-Aldrich or Matreya induced accumulation of lipids as demonstrated by quantification of Oil Red O staining. Docosapentaenoic acid and EPA from Sigma-Aldrich did not induce lipid accumulation, while DPA and EPA from Matreya did induce lipid accumulation (Fig. 1C and D). Therefore, for all subsequent experiments only FFAs from Matreya were used. As a control of the solvents, USP grade ethanol at a 10 μl/ml concentration and cell culture grade DMSO at 1 μl/ml concentration were used without any effects on cell viability, lipid accumulation or expression of assessed proteins (data not shown).

### In contrast to other tested FFAs, ω-3 PUFAs selectively affect expression of adipose differentiation markers

**Figure 3:**
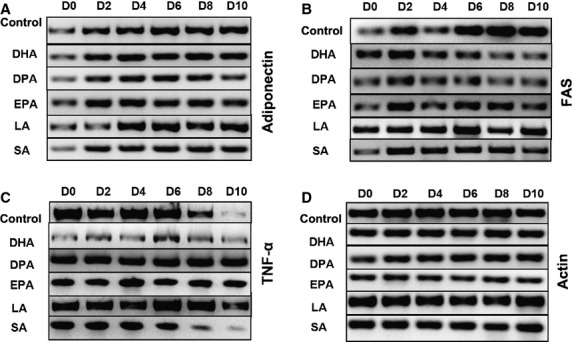
Semiquantitative RT-PCR showing the effects of a long incubation of 3T3-L1 adipocytes undergoing differentiation from D0 to D10 with the different free fatty acids. Gels are grouped by the same primer used, indicated on right side of the figures, while on the left side of the gels different treatments are indicated. Figures are representative of three experiments.

All three ω-3 PUFAs induced a significant rise in adipophilin levels throughout a 10-day differentiation course, which was much less pronounced with SA and LA (Fig. 2A). All three ω-3 PUFAs induced a sharp decline in SREBP1 and FAS protein levels starting at day 6 of incubation (Fig. 2B and C). All three ω-3 PUFAs as well as SA induced GLUT4 expression earlier than control or LA-treated cells (Fig. 2D). All three ω-3 PUFAs decreased the levels of PPARγ, but this effect was not specific to ω-3 PUFAs as it was observed with control LA and SA as well (Fig. 2K). None of the ω-3 PUFAs affected the levels of perilipin (data not shown). Induction of adiponectin mRNA observed during the course of adipose differentiation remained unaffected (Fig. 3A). As mentioned above, ω-3 PUFAs induced a decline in the levels of FAS protein, however levels of FAS mRNA remained stable (Fig. 3B).

### Omega-3 PUFAs decrease activity of the UPS

All three ω-3 PUFAs depressed the UPS, as demonstrated by accumulation of polyubquitinated proteins above control levels (Fig. 2E) associated with a decrease in levels of the β1 subunit of the 20S proteasome as well as S4 subunit of the PA700 proteasome activator (Fig. 2F and G). Stearic acid and LA did not affect tested UPS markers.

### Omega-3 PUFAs do not induce apoptosis or cellular stress

Omega-3 PUFAs from Sigma-Aldrich induced apoptosis and cellular stress, however those effects likely depended on the presence of uncharacterized contaminants (data not shown). Omega-3 PUFAs from Matreya did not induce caspase-3 cleavage (Fig. 2H). Levels of other tested markers, such as VCP, VIMP, CHOP, JNK, or phosphorylation of JNK remained stable with all FFAs tested throughout the entire differentiation course (data not shown). There was a rise in the ER chaperone BiP associated with adipose differentiation in control cells, which was unaffected by any treatment (Fig. 2I). This BiP induction likely represents ER stress.

### Omega-3 PUFAs modulate the expression of inflammatory markers

There is an induction of NFκB during adipose differentiation, which becomes more prominent at day 6 (Fig. 2J). SA as well as all three ω-3 PUFAs do not interfere with NFκB induction. However, LA suppressed NFκB induction. Tumour necrosis factor-α levels decline during adipose differentiation, starting at day 8 as assessed by semiquantitative RT-PCR (Fig. 3C). All three ω-3 PUFAs as well as LA stabilized levels of the TNF-α mRNA, while SA had no effects.

### FAS degradation induced by ω-3 PUFAs is mediated by the UPS

As mentioned before, all three ω-3 PUFAs, but not SA or LA, induce a significant decline in FAS expression starting at day 6 of the incubation (Fig. 2B). Semi-quantitative RT-PCR showed that FAS mRNA levels remained stable (Fig. 3B), suggesting that the decline in FAS protein levels is a post-translational process.

**Figure 4:**
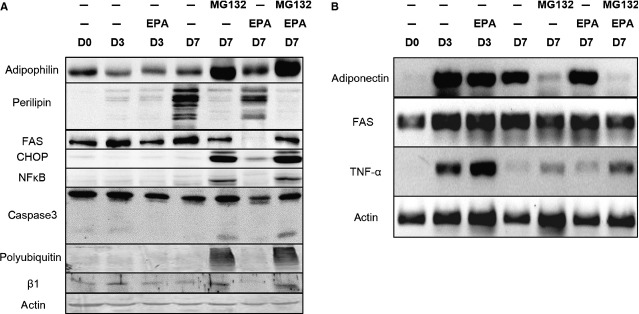
Western blots (A) and semiquantitative RT-PCR (B) showing the effects of EPA alone or in combination with the proteasome inhibitor MG132 on 3T3-L1 on adipocytes incubated for 3 days: day 3 till day 7 of the differentiation course. Figures are representative of three experiments.

In order to test whether FAS degradation depends on the UPS, we have incubated day 3 adipocytes differentiated in the presence of EPA since day 0, for additional 4 days with EPA and the proteasome inhibitor MG132 (Fig. 4). While EPA induced degradation of FAS protein as seen on Western, FAS mRNA levels were unaffected as seen by semiquantitative RT-PCR. The proteasome inhibitor MG132 prevented the EPA-induced degradation of FAS protein, without any effect on FAS mRNA levels. Omega-3 PUFAs therefore appear to induce UPS-dependent degradation of FAS.

### Anti-adipogenic effects of proteasome inhibition

We also wanted to explore further the effects UPS may have in adipose differentiation. As expected, proteasome inhibition led to accumulation of polyubiquitin and a compensatory up-regulation of the β1 subunit of the 20S proteasome (Fig. 4A). While visual inspection did not show any prominent cell death, Western analysis revealed induction of CHOP and partial caspase-3 cleavage consistent with induction of apoptosis. MG132 induced adipophilin much stronger than EPA, while at the same time it led to an almost complete down-regulation of perilipin (Fig. 4A) and adiponectin mRNA (Fig. 4B). At the same time, it up-regulated NFκB and TNF-α expression (Fig. 4A and B).

### Combination of ω-3 PUFAs with different inhibitors

**Figure 5:**
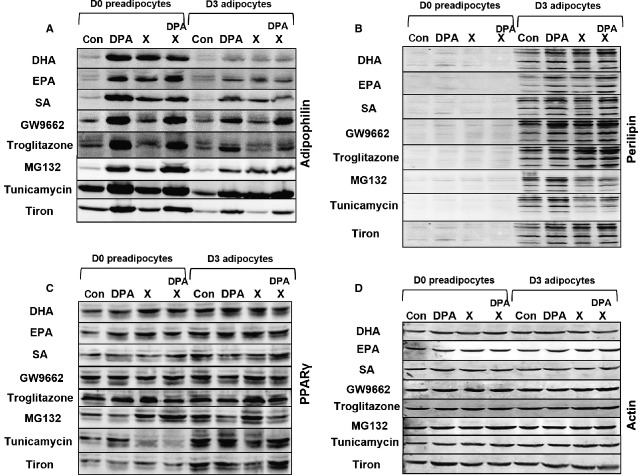
Western blots showing the effects of a combination of docosapentaenoic acid with various free fatty acids as well as with indicated inhibitors on day 0 pre-adipocytes or day 3 adipocytes derived from 3T3-L1 fibroblasts. Westerns are grouped by the same antibody used indicated on the right, while different treatments are indicated on the left of each figure. X indicates that the compound listed on the left side of the blot has been used. Figures are representative of three experiments.

To elucidate the mechanisms mediating the effects of ω-3 PUFAs, we have incubated day 0 pre-adipocytes or day 3 adipocytes for 24 hrs with DPA in combination with other FFAs as well as with selected inhibitors of relevant pathways. We analysed the effect of this treatment on the expression of three important adipogenic markers: adipophilin, perilipin and PPARγ (Fig. 5).

Docosapentaenoic acid alone induced adipophilin in both day 0 pre-adipocytes and day 3 adipocytes, did not affect PPARγ expression in either day 0 pre-adipocytes or day 3 adipocytes, and only slightly induced perilipin expression in day 3 adipocytes (in day 0 pre-adipocytes, perilipin levels were below detection threshold). Combination of DPA with DHA, EPA or SA to a total FFAs concentration of 200 μM did not affect the effects observed with DPA alone.

Subsequently, we explored whether the pro-adipogenic PPARγ transcription factor is involved in the effects of ω-3 PUFAs. Neither the PPARγ agonist troglitazone, nor the PPARγ antagonist GW9662 interfered with DPA-induced up-regulation of adipophilin in day 0 and 3 cells. Both troglitazone and GW9662 induced perilipin in day 3 cells, regardless of the presence of DPA. While GW9662 did not affect PPARγ levels, troglitazone induced PPARγ in both day 0 and day 3 cells, an effect which appears to be antagonized by DPA.

As ER stress is associated with adipogenesis, we decided to investigate whether induction of ER stress by two different methods interferes with the effects of ω-3 PUFAs. Proteasome inhibitor MG132 and N-glycosylation inhibitor tunicamycin induced adipophilin up-regulation in both day 0 and day 3 cells, at the same time down-regulating perilipin expression in day 3 cells regardless of the presence of DPA.

Free radicals may mediate at least some of the effects of -3 PUFAs. Therefore, we decided to test whether the free radical scavenger Tiron affects the effects exerted by DPA. We have not observed any interference with DPA effects when Tiron was added. When used alone, it did not affect expression of any of the tested adipose markers.

## Discussion

Altogether, our results indicate that ω-3 PUFAs have beneficial metabolic effects on adipose cells and promote cell growth. Reported antiproliferative or pro-apoptotic effects of ω-3 PUFAs 22,26 can be likely attributed to toxic effects of impurities present in reagents. It is generally assumed that ω-3 PUFAs increase lipolysis and decrease lipogenesis through several pathways 26–29. However, in other reports, EPA has been shown to inhibit lipolysis 30,31. We have observed increased accumulation of neutral fats within 24–48 hrs of incubation with all three ω-3 PUFAs. Another measure of pro-adipogenic effects of ω-3 PUFAs was the induction of adipophilin, an adipogenic protein associated with lipid droplets at their early stage of formation. Observed induction was specific to ω-3 PUFAs. As lipid droplets coalesce and mature, they are associated with perilipin, which protects them from lipolysis. None of the ω-3 PUFAs affected the levels of perilipin, contrasting with reports that DHA decreased perilipin expression 28. While those findings indicate pro-adipogenic effects of ω-3 PUFAs, some anti-adipogenic effects were observed as well. For example, all three ω-3 PUFAs induced a sharp decline in FAS and SREBP1 expression, consistent with reports in other systems 3,32. None of ω-3 PUFAs affected PPARγ and adiponectin when compared to the control FFAs LA and SA, while others reported either a ω-3 PUFA-specific induction of PPARγ 33 and adiponectin 33–36, or on the contrary, suppression of PPARγ 37 and adiponectin 38 upon treatment with ω-3 PUFAs. SA and all three ω-3 PUFAs induced the insulin sensitive glucose transporter GLUT4, paralleling findings in muscle fibres 39.

At least some effects of ω-3 PUFAs are believed to depend on their interaction with PPARγ 34,35,40, but we have not observed any interference with their effects by co-incubation with its agonist or antagonist. Docosapentaenoic acid appeared, however, to antagonize induction of PPARγ by troglitazone that can be explained by the fact that DPA is a much weaker, partial agonist of PPARγ than troglitazone.

Obesity is associated with a low level of chronic inflammation 41. While saturated fatty acids promote inflammation, beneficial ω-3 PUFAs effects involve modulation of the inflammatory response 8. We have observed an induction of NFκB during adipose differentiation, which was not affected by ω-3 PUFAs or SA. Only LA had anti-inflammatory effects. Declining TNF-α mRNA levels during adipose differentiation were stabilized by LA and ω-3 PUFAs. While LA is known to induce this cytokine 42, no ω-3 PUFA-mediated TNF-α induction was ever described. Apparent lack of anti-inflammatory effects of ω-3 PUFAs can be explained if they are mostly indirect through adipose tissue macrophages, absent in our system 43.

Accumulation of polyubiquitinated proteins induced by all three ω-3 PUFAs in adipocytes is a novel effect, which appears to be mediated by down-regulation of proteasomal subunits. A similar effect was seen in muscles of cachectic mice 44 and human endothelial cells 45. We have observed for the first time a selective UPS-dependent FAS degradation induced by ω-3 PUFAs in adipocytes, resembling selective degradation of oestrogen receptors 46 or β-catenin 47 in other systems. Omega-3 PUFAs can affect the ubiquitinating enzymes as well 48. Identification of mechanisms mediating effects of ω-3 PUFAs on different components of the UPS in adipocytes will identify new targets for therapy of obesity and metabolic syndrome.

Proteasome inhibition in adipocytes led to accumulation of polyubiquitin and a compensatory up-regulation of proteasome subunits accompanied by accumulation of adipophilin, which is normally degraded in lipid-poor conditions by the UPS 40. However, down-regulation of perilipin, likely to be mediated by the lysosomal pathway 49, indicates that proteasome inhibition may actually promote lipolysis and/or prevent lipogenesis. This is further supported by down-regulation of adiponectin mRNA. Adipocytes appeared surprisingly resistant to the pro-apoptotoic effects of proteasome inhibition 50, but prolonged incubation led to induction of some apoptosis markers. At least some of the effects of proteasome inhibition may be mediated by ER stress and unfolded protein response, known to be involved in adipose differentiation 51. Indeed, tunicamycin induced adipophilin up-regulation and perilipin down-regulation very similar to proteasome inhibition.

In conclusion, we have shown that ω-3 PUFAs exert a complex effect on adipocytes, with both anti-adipogenic and pro-adipogenic components, however they do not induce cell death. An important mechanism of action appears to be modulation of the UPS, accompanied by accumulation of polyubiquitinated proteins, down-regulation of proteasome subunits and selective degradation of FAS, and possibly other, yet unidentified proteins.
